# A Sensory Shelf-Life Study for the Evaluation of New Eco-Sustainable Packaging of Single-Portion Croissants

**DOI:** 10.3390/foods13091390

**Published:** 2024-04-30

**Authors:** Roberta Tolve, Lucia Sportiello, Giada Rainero, Andrea Pelattieri, Marco Trezzi, Fabio Favati

**Affiliations:** 1 Department of Biotechnology, University of Verona, Strada Le Grazie 15, 37134 Verona, Italy; lucia.sportiello@univr.it (L.S.); fabio.favati@univr.it (F.F.); 2 Research and Development Department, Bauli S.p.A., Via Giuseppe Verdi 31, 37060 Castel D’Azzano, Italy; giada.rainero@bauli.it (G.R.); andrea.pelattieri@bauli.it (A.P.); marco.trezzi@bauli.it (M.T.)

**Keywords:** Avrami model, croissant, eco-packaging, sensory analysis, shelf-life, survival analysis

## Abstract

Understanding the correlation between straightforward analytical methods and sensory attributes is pivotal for transitioning to sustainable packaging while improving product quality. In this context, the viability of eco-sustainable packaging alternatives for single-packaged croissants has been investigated through examining the correlations between analytical methods, sensory attributes, employing quantitative descriptive analysis (QDA), and consumer survival analysis. The performance of biaxially oriented polypropylene (BOPP), a petrochemical plastic film, against paper-based, compostable, and biodegradable films over a 150-day croissant storage period was compared in this study, examining both physiochemical and sensory perspectives. The results showed a correlation between a lower water vapour barrier in packaging materials and increased moisture migration and croissant hardness, as assessed by the Avrami kinetic model. Notably, given its reduced barrier properties, the compostable film accelerated sensory profile deterioration, as evidenced by QDA results. Shelf-life estimation, assessed by consumer rejection, underscored the viability of the biodegradable film for up to 185 days, surpassing BOPP, paper-based, and other biodegradable alternatives. Using linear regression, physiochemical parameters associated with predicted shelf-life were elucidated. Overall, croissants were rejected by 50% of consumers when they reached humidity levels below 18%, water activity below 0.81, firmness exceeding 1064 N, pH above 4.4, and acidity below 4.5. Based on the results of this study, biodegradable packaging emerges as a promising alternative to traditional BOPP, offering a sustainable opportunity to extend the shelf-life of croissants.

## 1. Introduction

Bakery products, such as croissants, are popular breakfast items consumed in many parts of the world. Croissants are fermented layered bakery products made from flour, salt, water, yeast, shortening, sugar, egg, non-fat milk powder, and margarine or butter. The main factors that can alter croissants are staling, related to moisture migration from the product to the environment, and fat oxidation and spoilage caused by bacteria, yeast, and moulds in products with water activity >0.85 [1,2,3]. Due to their high purchase frequency and turnover, single-portion packed croissants, classified as fast-moving consumer goods, contribute significantly to the consumption of plastics and other non-biodegradable materials [4]. Currently, biaxially-oriented polypropylene (BOPP), a petrochemical plastic film, is an extensively used packaging in the industry because it is relatively cheap, has good tensile properties, and represents an effective barrier against water vapour [5]. BOPP is not biodegradable, but is technically recyclable. In actuality, even if it is correctly delivered to a suitable facility at the end of its life, it does not return to its original packaging function, thus interrupting effective circularity [6]. Consequently, due to the serious environmental concerns about this synthetic film and the interest of consumers in safe food products with longer shelf lives, an increasing level of attention has been paid to eco-friendly packaging with the desired physical, mechanical, and barrier properties needed for food packaging [7,8]. However, modifying the packaging of croissants involves reassessing the product’s shelf-life since, as with every packed product, the croissants’ shelf-life depends on the packaging [9]. Because of this, selecting proper alternative packaging that can prevent chemical, physical, and sensory deterioration and is capable, at the same time, of lengthening shelf-life and increasing croissant quality is crucial for food companies.

Shelf-life is defined as the time during which a product retains its safety and adheres to its quality specifications under standard conditions of storage, distribution, and utilization [10,11]. Comparatively, sensory shelf-life describes the period over which a product maintains its intended sensory characteristics and performance, as the manufacturer specifies. It can be estimated through various sensory testing methods, including discriminant, descriptive, and affective methodologies [12,13]. These methods involve monitoring the intensity of specific attributes during food storage, allowing for the estimation of a food’s shelf-life when these intensities reach a predefined threshold [14]. While sensory testing methods have been emphasized in studies on food shelf-life, producers are increasingly seeking rapid and non-destructive techniques [15]. Since, as mentioned above, the sensory attributes of croissants play a significant role in determining their overall appeal and, consequently, their shelf-life, establishing correlations between straightforward analytical methods and variations in critical sensory attributes offers a practical and valuable tool in managing the switch versus eco-sustainable packaging. Considering the context provided above, this study aimed to evaluate the shelf-life of single-portion croissants sealed in different eco-sustainable packaging materials by combining straightforward analytical methods and multivariate analysis, as well as to define a possible correlation between sensory profile, consumer product rejection, and analytical methods. In detail, croissants were sealed in standard BOPP plastic film and three eco-sustainable alternatives: a paper-based/PP coextruded film, a compostable PLA Met film (Poly lactic acid metalized)/Mater-Bi, and a biodegradable BOPP film modified with a masterbatch to facilitate biodegradation without producing microplastics or quality loss. The physiochemical and sensory profiles of individually packaged croissants stored for 150 days were assessed. 

## 2. Materials and Methods

### 2.1. Standards, Reagents and Solvents

Sucrose (purity > 99.5%), sodium chloride (purity > 99%), caffeine (purity > 99%), citric acid monohydrate (purity > 99.5%), and glutamic acid monosodium salt monohydrate (purity > 98%) used to investigate taste function for the five basic tastes were purchased from Merck (Vimodrone, Milano, Italy).

All other chemicals were of analytical grade or equivalent and were obtained from Merck (Vimodrone, Milano, Italy). Ultrapure water was obtained from a Milli-Q system (Millipore, Billerica, MA, USA).

### 2.2. Croissant Production

Croissant production was carried out at Bauli SpA (Verona, Italy) using the following ingredients: wheat flour, vegetable margarine (palm fat, water, sunflower oil), natural yeast, sugar, fresh eggs (7.5%), glucose-fructose syrup, surface crystalline sugar, high-quality fresh pasteurized milk (1.5%), and emulsifiers: mono-and diglycerides of fatty acids, butter, salt, flavourings, pea protein, and dextrose. The ingredients were mixed evenly during the kneading stage. The dough was allowed to rest for one minute. Then, after the dough was spread, three sequential rolling steps were performed with the rolling grease to achieve flaking in the final product. The final dough was cut into triangular shapes, which were then rolled to form croissants. The resulting forms were placed in baking pans to proceed to the leavening stage (6 h). Samples were baked (15 min at 195 °C) in an industrial oven and cooled before packaging. An alcoholic flavouring solution was sprayed during the packaging stage to enable product preservation.

### 2.3. Packaging Material

The obtained croissants were single-packaged. Based on the type of primary packaging, the samples were named plastic (standard packaging in BOPP), paper based (paper/PP coex met), compostable (PLA Met/Mater Bi), and biodegradable (BOPP + masterbatch). Table 1 describes the packaging material’s description concerning stratigraphy, thickness, water vapour transmission rate (WVTR), and oxygen transmission rate (OTR). All croissants were then packaged into standard secondary cardboard box packaging.

### 2.4. Storage Conditions

Packaged croissants were stored under controlled temperature and moisture conditions (21 °C +22% RH). Changes in the characteristics of croissants sealed with four different films were evaluated after 7, 30, 60, 90, 120, 130, 140, and 150 days following the production day.

### 2.5. Moisture and Water Activity 

Croissant samples were ground for 30 s to perform moisture content and water activity (a_w_) analysis. 

Croissant moisture content was measured based on 5 g of the sample using a ventilated oven set at 105 ± 2 °C until a constant weight was achieved. The moisture content was then calculated as follows (Equation (1)):

(1) $$\mathrm{Moisture}\;(\%)=\frac{the\;weight\;of\;the\;fresh\;sample\;\left(g\right)\;-\;the\;weight\;of\;the\;dried\;sample\left(g\right)}{weight\;of\;fresh\;sample\left(g\right)}$$

Water activity (a_w_) was measured using the Hygropalm HC2-AW analyser (Rotronic Italia Srl, Milano, Italy) at 25 °C. Samples were analysed in triplicate.

### 2.6. Total Titratable Acidity and pH

Ten grams of ground sample was mixed with 250 mL of distilled water and homogenized using an Ultraturrax T10 (IKA-Werke GmbH & Co. Staufen, KG—Germany) (12,000 rpm for 30 s). The pH was measured by holding the sample in agitation with a magnetic stirrer and using a pH meter (Hanna Instruments Ltd., Bedfordshire, UK).

The total titratable acidity of the samples was assessed. Titration using 0.1N KOH solution from the homogenized sample, as described above, was carried out until a pH of 8.3 was reached. The titratable acidity was expressed in milli equivalent alkali.

### 2.7. Texture Analysis of Croissant

Croissants were analysed using a TA. XT-plus Texture Analyser (EN.CO S.r.l., Venice, Italy). The probe used for firmness determination was the SMSP/50 mm, and the following parameters were used: 40 mm return distance, 10 mm/s return speed, and 1 g contact force. After cutting the sample in half, each half had the end removed, obtaining two 3 cm-wide sections. The width corresponded to the height of the sample to be inserted under the probe. The firmness value was expressed in Newton (N).

The variation of firmness with time was used to describe croissant staling (caused by starch retrogradation) using the equation of Avrami (Equation (2)):

(2) $$\Theta =\frac{F\infty -Ft}{F\infty -F0}=e^{{-kt}^{n}}$$

where Θ is the fraction of uncrystallized material at time t expressed in terms of firmness (dimensionless); F_0_ is the firmness (in g.force) of the fresh croissant; F_t_ is the firmness (in g.force) at time t; F_∞_ is the limiting value of firmness (g.force) at 150 days of storage; k is the rate constant (per day); n is a constant, and n is the Avrami exponent relating to geometry and nucleation type and provides qualitative information on the nature of crystal growth.

Equation (2) can be alternatively expressed as:

(3) $$\log\;(-\ln\;\frac{F\infty -Ft}{F\infty -F0})=\log k+n\log\;t$$

Accordingly, the n and K values can be obtained from the slope and intercept of log (−ln Θ) versus log t plot. The crystallization half-time (t_1/2_) is the time at which the extent of crystallization is half-completed (50%). It can be determined from the measured kinetic parameters k and n (Equation (4)):

t_1/2_ = (ln 2/k) ^(1/n)^ (4)

Shorter crystallization half-time is associated with a faster crystallization rate [16].

### 2.8. Sensory Evaluation

Quantitative descriptive analysis (QDA) was conducted utilizing a trained sensory panel comprising thirteen members (six females and seven males) aged between 24 and 46 years, recruited from the University of Verona. Assessors were carefully selected and trained. The panel underwent a three-month training period, focusing on 17 descriptive terms that defined themselves. 

This sensory vocabulary was developed through panel discussions and encompassed attributes related to croissants’ appearance, odour, flavour, taste, texture, and mouthfeel (Table 2). Reference standards were established for each descriptor, corresponding to the highest intensity score on the rating scale.

After calibrating the panel, they assessed the four croissant samples at 7, 30, 60, 90, 120, 130, 140, and 150 days from the production date. Sensory evaluations occurred in individual booths under controlled light, temperature, and humidity conditions, with samples served on white ceramic plates. The samples, labelled with three-digit codes, were presented among the subjects in a completely randomized and balanced order. Each session involved two replicates, during which the assessors rated the croissant samples based on the selected attributes using a predefined scale (0–100), where a higher score indicated a higher intensity of the given sensory attribute. Before tasting each sample, panellists were instructed to cleanse their palates with distilled water. Sensory sessions were conducted using FIZZ V2.51 software (BioSystem, Couternon, France). 

Furthermore, 40 consumers were surveyed for each storage time, responding to a binary question: ‘Would you typically consume this product?’ The responses, categorized as ‘yes’ or ‘no’, were then utilized in survival analysis methodology to determine the shelf-life of croissants packed in various types of packaging. Informed written consent according to the principles of the 1964 Helsinki Declaration and its subsequent revisions was obtained from all the participants before the test. Furthermore, we adhered to Regulation (EU) 2016/679 of the European Parliament of the Council dated 27 April 2016 [17] concerning personal data safeguarding and the unimpeded flow of such information involving natural persons.

### 2.9. Statistical Analysis

The physiochemical analyses were carried out at least in triplicate, and the mean values ± standard deviation were reported. The variables were tested for significance using a two-way analysis of variance (ANOVA). Differences among means were assessed using Tukey’s HSD tests (*p* < 0.05). 

Regarding the QDA, throughout the entirety of their training, the panel was calibrated through obtaining the mean rating. Panellists whose ratings were not within 10% of the mean rating were asked to re-evaluate the samples and references and adjust their ratings until a consensus was reached. Panellists’ reproducibility was determined using analysis of variance (ANOVA) at *p* = 0.05 in the Panel Analysis tool in XLSTAT (Premium version 2019.4.2, Addilnsoft, SARL, Paris, France). Panellists whose ratings were not reproducible were assisted to improve performance. At the end of the descriptive analysis, the Panel Analysis tool was used to assess the panellists’ consensus, the discrimination ability of the descriptors, and whether there were assessor and session effects. F values, mean squared error (MSE) values, and p x MSE were used to assess the ability of sensory evaluators. In the subsequent analysis, only the descriptors that were significantly (*p* < 0.05) discriminated were included. Pearson’s correlation coefficient and a multivariate analysis approach were applied to the sensory attribute scores. Using the Pearson correlation matrix, a principal component analysis (PCA) was conducted in order to investigate the relationships between the different packaging materials and the sensory characteristics of the croissants. The shelf-life of the product was analysed using data obtained from the survival analysis. The cutoff was established by the Weibull distribution, considering a 50% probability of acceptability by the panellists. Figures were generated using Graphpad Prism 8 software (Graphpad Software, San Diego, CA, USA). A correlogram plot was created using R Studio (RStudio version 2022.07.1, Inc., Boston, MA, USA).

## 3. Results and Discussion

### 3.1. Croissant Physiochemical Characterization

Croissants sealed in plastic, paper based, compostable and biodegradable film were analysed over 150 days to assess the impact of packaging and storage time on the product’s characteristics. 

The moisture content of baked products is a crucial factor that significantly influences their overall quality, affecting factors such as microbial growth, organoleptic degradation, functional and structural properties, non-enzymatic browning, lipid oxidation, textural changes, and aroma retention. Throughout the storage period, the moisture content of all of the samples gradually decreased, leading to changes in the product’s quality (Figure S1). Notably, the freshly baked croissants exhibited the highest moisture content at time 0, which then decreased over time. A similar trend was observed for the water activity in all of the samples. Water activity is an essential parameter often used to determine the critical moisture content necessary to ensure mould-free shelf-life and organoleptic acceptance of baked products. The data indicated that over the 150 days of storage, the average reduction in moisture content was 9.6%, and the reduction in water activity was 5.3% compared to the initial values for the croissants. However, this decrease was not consistent across the different packaging materials used. Notably, the compostable film, made with metallized PLA and Mater-Bi, exhibited the worst preservation performance in terms of moisture loss, with a reduction of up to 15.8% for moisture and 9.9% for water activity. The standard plastic and biodegradable films preserved the croissants better, with a loss of 6.8% and 6% of moisture and 2.7–4% of water activity, respectively. Generally, as also reiterated by Wang et al. [18], packaging materials with lower WVTR are better at inhibiting water loss from packaged foods. Therefore, selecting packaging with higher gas barrier properties can effectively extend the shelf-life of products. 

Decreases in moisture level are one of the main factors responsible for quality reductions in baked goods. Specifically, a loss of moisture produces hard and dry crumbs in croissants, and, as reported by Patel et al. [19], avoiding this phenomenon during long-term storage is difficult. Le-Bail et al. [20] observed that the decline in moisture observed during the storage of baked goods correlates with the progress of starch retrogradation and crumb staling, also noting a decrease in the water-holding capacity of starch during baked goods storage. Notably, in the 150 days of storage, there was a simultaneous increase in the sample firmness, a crucial textural parameter for bakery goods (Figure S1). According to Kwaśniewska-Karolak and Mostowski [21], these changes are significantly influenced by storage conditions and the type of packaging material used [21]. Our findings revealed that the single-packed croissants were highly susceptible to textural changes during storage. Croissants packaged with compostable material exhibited a higher increase in firmness, with a final value of 1516 N, three times the initial value. Similar trends were observed for croissants packaged with other films, with values consistently below 1092 N.

The increase in firmness in bakery products is a complex process, primarily related to starch retrogradation, but not limited to it. Other processes, such as water transfer, colloidal solubility, and the degradation of components like lipids or proteins, also play a role [22]. The acidity of bakery products generally has an impact on the crumb texture, flavour, and resistance to the effects of harmful aerobic bacteria. Wheat products are characterized by low acidity; for the croissants at time 0, the acidity was 3.6. This value was consistent with the findings of other studies [21]. The acidity slightly increased over the entire storage period, up to 4. The pH value and the acidity of the croissant showed a slight tendency to decrease and increase, respectively, across the 150 days of storage. As can be observed from Table 3, there was a significant effect relating to the interaction between packaging and storage time only for moisture, water activity, and firmness, indicating that different packaging materials preserve these croissant parameters differently over time. Specifically, a more significant decrease in moisture and water activity was observed for the compostable film, while the biodegradable film exhibited the opposite trend. Simultaneously, the compostable film resulted in a significantly higher increase in firmness, whereas the biodegradable film showed the opposite trend. The lower vapour barrier properties of the compostable film could also explain these findings.

### 3.2. Kinetic Model on Staling of Croissant during Storage

The staling process in bakery products is a complex phenomenon influenced by multiple factors. One primary indicator of staling during storage is an increase in firmness, typically caused by water loss and starch retrogradation [23]. Over the 150 day storage period, the croissants consistently exhibited a significant increase in firmness, regardless of the type of packaging used. This increase can be attributed to starch retrogradation, a process involving the crystallization of starch molecules initiated by spontaneously formed nuclei, catalysing the growth of additional crystals. The Avrami equation (Equation (1)) accurately describes this phenomenon. Table 4 summarizes the study’s findings, presenting various kinetic parameters such as F_∞_ (final firmness), t_1/2_ (crystallization half-time), n (Avrami exponent), k (rate constant), and R^2^. These parameters were pivotal in distinguishing the staling kinetics of croissants packaged with different films during storage. Notably, the firming kinetics of croissants were strongly influenced by the values of n and k. Higher values of n or k corresponded to rapid firming kinetics, while lower values of these parameters and reduced F_∞_ values indicated slower firming kinetics [24]. It is worth noting that the exponent ‘n’-calculated values consistently exceeded 1 (n > 1) across all samples. This suggests, in line with the findings of a previous study by Campo et al. [25], that amylopectin crystals within the croissants grew rod-like from sporadic nuclei. Of particular interest, croissants packaged with the compostable film displayed higher rate constant (k) values, resulting in a firmer product with accelerated staling kinetics during storage. Conversely, croissants packaged with biodegradable film demonstrated lower F_∞_ values and a reduced Avrami exponent (n). Considering these outcomes, selecting the biodegradable film for single-packed croissants could extend the product’s softness over time.

### 3.3. Sensory Analyses

The results of the QDA (quantitative descriptive analysis) sensory analysis, which examined the impact of packaging material and storage time, as well as their interaction, on the sensory profile of croissants, including attributes related to appearance, odour, flavour, taste, texture, and mouthfeel, are presented in Table 5. Both packaging and time factors significantly influenced most of the attributes evaluated by the panel. On the other hand, their interaction significantly affected only structural attributes, softness, elasticity, stickiness, consistency, and softness in the mouth. During the storage period, noticeable changes were observed (Table S1). As expected, there was a significant decrease in overall odour, vanilla odour, and butter odour, accompanied by an increase in alcohol odour. This trend aligns with findings from previous studies examining sensory attributes across the shelf-life of bakery products [15,26,27]. This decline is attributed to packaging permeability, which allows these compounds to dissipate.

Additionally, these changes might be related to the formation of inclusion complexes with amylose, as hypothesized by Licciardello et al. [26] in their assessment of the shelf-life of industrial durum wheat bread within different packaging systems. Specifically, the compostable film emerged as the least effective in preserving the croissants’ olfactory characteristics. Similarly, there was a reduction in sweetness, overall flavour, butter flavour, and vanilla flavour over time. Texture attributes, such as softness and elasticity evaluated by touch and softness evaluated by taste, displayed a diminishing trend during storage, accompanied by increased consistency. The variation in these attributes appeared to be influenced by the amount of water migrating from the product to the environment. Even for these descriptors, the variation over time was observed in all croissants, regardless of their packaging, with the compostable film found to be the least effective at preserving the products. The results from the sensory evaluation of croissants sealed with the four types of primary packaging corroborate and support the data obtained from instrumental analyses.

**Figure 1:**
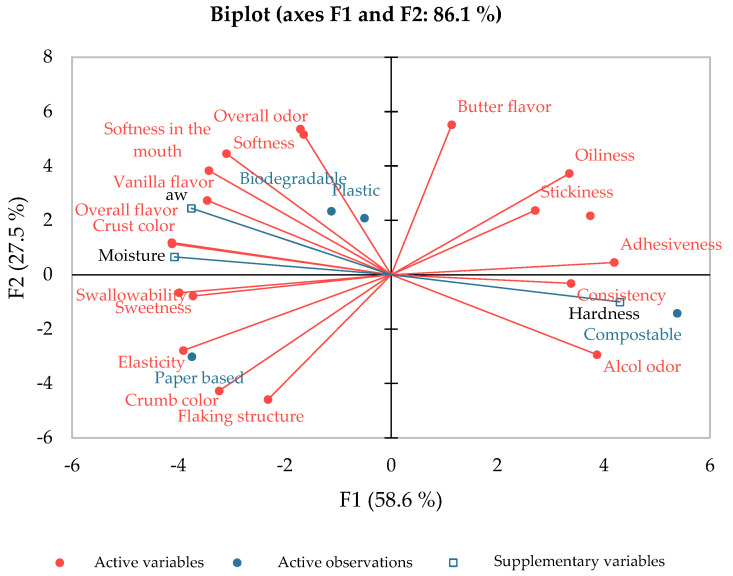
Principal component analysis investigating the relationships between the different packaging materials (plastic, paper based, biodegradable, and compostable) and the sensory croissant descriptors. Additionally, water activity, moisture and hardness were used as supplementary variables for interpretative purposes.

In order to investigate the relationships between the different packaging materials and the sensory croissant descriptors, a principal component analysis was developed. With this objective in mind, the sensory characteristics and the different types of packaging were used as active variables; the chemical-physical properties, such as water activity, moisture, and hardness, were used as supplementary variables for interpretative purposes. In Figure 1, the biplot analysis of the principal components is reported. The first two components (PC1 and PC2) extracted explained 58.6% and 27.5% of the variability, respectively, for a total of 86.1%. The PCA biplot revealed a clear clustering of the croissants sealed with different packaging. The second quadrant contains the samples packaged using the plastic and biodegradable packaging, which were characterized by better olfactory and flavour characteristics at the end of the shelf-life. In contrast, the third quadrant contains the samples packaged with the paper based film, which were characterized by a generally sweeter taste and higher elasticity. Lastly, the fourth quadrant contains the croissants packaged with the compostable film, characterized by a greater perception of alcohol odour, probably due to a reduced perception of all of the other olfactory attributes and greater hardness, observable both from the sensory attribute consistency and from the supplementary variable firmness. Being located in opposite quadrants, croissants packaged with biodegradable and plastic films were characterized by reduced consistency, reduced smell of alcohol, and a higher level of humidity and water activity.

### 3.4. Correlation Coefficients between Variables

**Figure 2:**
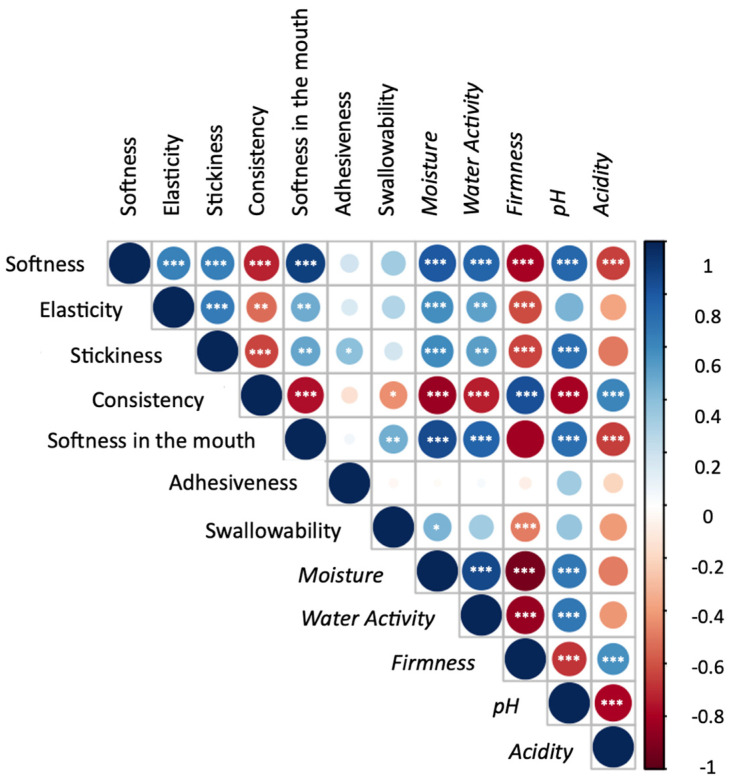
Correlogram between sensory attributes for which a significant effect of the storage and packaging material have been considered, along with the physiochemical parameters (*italics*) of the croissant. Colours indicate different correlation coefficient values according to the scale bar reported. The size of the circle is proportional to the correlation coefficients. Asterisks indicate the significance of the Pearson correlation coefficient (*, **, *** correspond to *p* ≤ 0.05, 0.01. and 0.001, respectively).

The correlogram of the physiochemical and sensory variables under investigation in this study is shown in Figure 2. Specifically, only the sensory attributes for which the storage and packaging material had a significant impact were considered. In summary, all the sensory attributes evaluated, except for adhesiveness, exhibited significant correlations with the straightforward physical and chemical parameters investigated. Moisture and water activity in croissants showed a positive correlation with softness (both by touch and in mouth), elasticity, stickiness, and, to a lesser extent, swallowability. Conversely, they were inversely correlated with consistency. Firmness showed the opposite trend for these attributes. It is worth noting that pH and acidity also exhibited contrasting trends. pH was positively correlated with softness, stickiness, and elasticity, while it was inversely related to consistency. These results suggest that predicting softness, elasticity, stickiness, and consistency through measuring physiochemical parameters is feasible.

### 3.5. Survival Analysis and Shelf-Life Prediction

**Figure 3:**
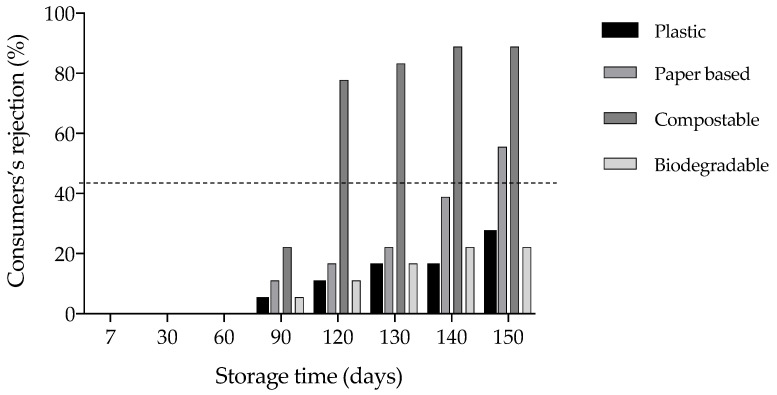
Consumers’ rejection of croissants packed with plastic, paper-based, compostable, and biodegradable film at each storage time. The dashed line corresponds to the 50% rejection rate used to define the shelf-life.

Survival analysis was employed to assess the shelf-life of croissants sealed using four different types of packaging. In this procedure, panellists gauged the products’ acceptability throughout storage by responding to the query, ‘Would you consume the sample?’ (answer options: yes or no). The key concept was to focus the shelf-life estimation on the risk of product rejection and link the rejection % to the storage time. In Figure 3, the preference distribution function plot of the survival analysis with the percentile data of product rejection, namely the time at which a certain percentage of consumers would no longer purchase the product, is displayed.

Failure criteria were established at 50% consumer rejection levels, representing the usual cutoff points utilized in survival analysis [28]. Considering the time at which 50% of consumers would cease purchasing the product, disparities among the various packaging options examined can be discerned, as observed in Table 6. In accordance with the previously discussed results, the croissant sealed in the compostable film exhibited a shorter predicted shelf-life, amounting to 109 days. Conversely, the croissant sealed in the biodegradable film displayed a slightly extended shelf-life compared to the one packed in plastic film (185 versus 169 days respectively). Finally, the physiochemical parameters associated with the predicted shelf-life were determined using linear regression (Table 6). Croissants are typically rejected by 50% of consumers when they reach humidity levels below 18%, water activity below 0.81, firmness exceeding 1064 N, pH above 4.4, and acidity below 4.5.

## 4. Conclusions

This comprehensive analysis conducted on croissants sealed in different packaging materials highlights the pivotal role of packaging in maintaining product quality and extending shelf-life. By examining physiochemical parameters and sensory attributes, the elucidation of the interplay between packaging type, storage time, and product characteristics has been assessed. The potential for estimating croissants’ primary shelf-life using straightforward analytical methods correlated with critical sensory attributes is of particular significance. This capability is precious for companies seeking to transition to more sustainable packaging options while ensuring product integrity and consumer satisfaction. This approach could be adapted and extended to various food and non-food items. By understanding the critical sensory indicators and employing straightforward analytical methods, manufacturers could sift through various eco-friendly packaging solutions before selecting the ones that allow them to contribute to reducing environmental impacts while maintaining, or even improving, product shelf-life and consumer satisfaction. In the present research, biodegradable packaging has been shown to increase the shelf-life of croissants, potentially delivering a dual positive impact: reducing environmental footprint through adopting eco-friendly packaging, and minimizing food waste resulting from shorter product shelf-life. Insights gained from this study, applicable to the evaluation of other innovative and eco-friendly packaging solutions, reveal that croissants were considered unacceptable by 50% of consumers when they exhibited humidity levels below 18%, water activity below 0.81, firmness exceeding 1064 N, pH above 4.4, and acidity below 4.5.

## Figures and Tables

**Table 1:** Packaging material description.

Packaging	Plastic	Paper Based	Compostable	Biodegradable
Film stratigraphy	BOPP	PAPER + PPcoex met	PLA Met + MaterBi	BOPP
Film thickness	25 μm	25 g/m^2^ +8 μm	20 μm + 18 μm	25 μm
WVTR at 23 °C—85%rh g m^−2^ d^−1^	1.17	0.03	1.55	1.22
WVTR at 38 °C—90%rh g m^−2^ d^−1^	4.85	0.17	4.31	4.38
OTR at 25 °C—50%rh cm^3^/m^−2^ d^−1^	1340	14.2	3.09	1150

BOPP = biaxially oriented polypropylene; PPcoex met = coextruded metalized polypropylene; PLA Met = poly lactide acid metalized; WVTR = water vapour transmission rate; OTR = oxygen transmission rate.

**Table 2:** List of attributes and definitions developed by the sensory panel and used for the descriptive profiling of the croissants.

Attribute	Definition
Appearance	
Crust colour	Degree of brown colour by visual evaluation
Crumb colour	Degree of yellow colour by visual evaluation
Flaking structure	Attribute refers to the flaking of the internal structure due to the lamination process
Odour	
Overall odour	Overall odour intensity after full sample assessment
Alcohol odour	Alcohol odour by orthonasal evaluation
Flavour	
Overall flavour	Overall flavour intensity after full sample assessment
Butter flavour	Butter flavour intensity of the inner part of the croissant
Vanilla flavour	Vanilla flavour intensity of the inner part of the croissant
Taste	
Sweetness	Sweetness intensity by taste evaluation
Texture	
Elasticity	Ability to return to the initial shape after a compression between the forefinger and thumb
Softness	Compressive strength using the forefinger
Oiliness	Oiliness released by the sample to the touch
Stickiness	Stickiness released by the sample to the touch
Mouthfeel	
Consistency	Attribute related to the texture that indicates the force required to cut the product with the teeth
Softness in the mouth	Mechanical attribute relating to the texture that indicates the ease with which the product compresses between the tongue and the palate according to its alveolation
Adhesiveness	Mechanical attribute related to the work necessary with the tongue to detach a food glued to the palate and teeth
Swallowability	Attribute related to the number of needed chewies to swallow the sample in the mouth and evaluate

**Table 3:** Effect of packaging and storage time on croissant physiochemical parameters.

Parameter	Packaging	Storage Time (Days)								*p*-Value		
		7	30	60	90	120	130	140	150	Packaging (P)	Storage (S)	P × S
Moisture	Plastic	19.47 ± 0.18 ^A,b^	18.98 ± 0.35 ^B,b^	18.37 ± 0.18 ^C,b^	18.63 ± 0.39 ^D,b^	18.41 ± 0.23 ^D,E,b^	18.20 ± 0.11 ^E,F,b^	18.23 ± 0.13 ^E,F,b^	18.15 ± 0.07 ^F,b^	<0.001	<0.001	<0.001
	Paper based	19.47 ± 0.18 ^d^	19.09 ± 0.09 ^c^	18.78 ± 0.09 ^c^	17.62 ± 0.03 ^c^	17.79 ± 0.29 ^c^	17.43 ± 0.52 ^c^	17.32 ± 0.60 ^c^	17.57 ± 0.04 ^c^			
	Compostable	19.47 ± 0.18 ^c^	19.15 ± 0.03 ^d^	18.73 ± 0.07 ^d^	17.69 ± 0.33 ^d^	17.20 ± 0.07 ^d^	17.01 ± 0.35 ^d^	16.99 ± 0.17 ^d^	16.39 ± 0.38 ^d^			
	Biodegradable	19.47 ± 0.18 ^a^	19.33 ± 0.07 ^a^	19.27 ± 0.44 ^a^	18.96 ± 0.10 ^a^	18.35 ± 0.20 ^a^	18.31 ± 0.09 ^a^	18.37 ± 0.23 ^a^	18.29 ± 0.16 ^a^			
aw	Plastic	0.838 ± 0.01 ^A,a^	0.840 ± 0.01 ^B,a^	0.832 ± 0.00 ^C,a^	0.824 ± 0.00 ^D,a^	0.823 ± 0.01 ^D,E,a^	0.819 ± 0.00 ^E,a^	0.813 ± 0.00 ^F,a^	0.815 ± 0.00 ^F,a^	<0.001	<0.001	<0.001
	Paper-based	0.838 ± 0.01 ^b^	0.832 ± 0.00 ^b^	0.832 ± 0.00 ^b^	0.816 ± 0.00 ^b^	0.809 ± 0.01 ^b^	0.803 ± 0.00 ^b^	0.804 ± 0.00 ^b^	0.799 ± 0.00 ^b^			
	Compostable	0.838 ± 0.01 ^b^	0.824 ± 0.00 ^c^	0.816 ± 0.00 ^c^	0.800 ± 0.01 ^c^	0.805 ± 0.00 ^c^	0.796 ± 0.00 ^c^	0.760 ± 0.00 ^c^	0.755 ± 0.01 ^c^			
	Biodegradable	0.838 ± 0.01 ^b^	0.830 ± 0.00 ^b^	0.823 ± 0.00 ^b^	0.817 ± 0.00 ^b^	0.809 ± 0.00 ^b^	0.810 ± 0.00 ^b^	0.806 ± 0.00 ^b^	0.804 ± 0.00 ^b^			
Firmness	Plastic	425.34 ± 12.76 ^G,b^	606.26 ± 18.19 ^F,b^	805.31 ± 24.16 ^E,b^	897.55 ± 26.93 ^D,b^	1047.50 ± 31.43 ^b^	1047.99 ± 31.44 ^b^	1059.02 ± 31.77 ^B,b^	1092.16 ± 32.76 ^A,b^	<0.001	<0.001	<0.001
	Paper-based	425.34 ± 12.76 ^b^	690.08 ± 20.70 ^b^	876.46 ± 26.29 ^b^	986.26 ± 25.59 ^b^	1010.35 ± 30.31 ^b^	1000.47 ± 30.01 ^b^	1028.08 ± 30.84 ^b^	1061.88 ± 31.86 ^b^			
	Compostable	425.34 ± 12.76 ^a^	704.43 ± 21.13 ^a^	820.20 ± 24.61 ^a^	1419.66 ± 42.59 ^a^	1390.67 ± 41.72 ^a^	1442.56 ± 43.28 ^a^	1451.49 ± 43.54 ^a^	1516.37 ± 45.49 ^a^			
	Biodegradable	425.34 ± 12.76 ^c^	518.36 ± 15.55 ^c^	702.76 ± 21.08 ^c^	733.36 ± 22.00 ^c^	844.57 ± 25.34 ^c^	877.59 ± 26.33 ^c^	913.51 ± 27.41 ^c^	916.78 ± 27.50 ^c^			
pH	Plastic	4.93 ± 0.15 ^A^	4.86 ± 0.15 ^A^	4.80 ± 0.14 ^A,B^	4.69 ± 0.14 ^BC^	4.61 ± 0.14 ^C^	4.59 ± 0.14 ^C^	4.56 ± 0.14 ^C^	4.54 ± 0.14 ^C^	n.s.	<0.001	n.s.
	Paper-based	4.94 ± 0.15	4.95 ± 0.15	4.91 ± 0.15	4.78 ± 0.14	4.67 ± 0.14	4.66 ± 0.14	4.63 ± 0.14	4.59 ± 0.14			
	Compostable	4.95 ± 0.15	4.84 ± 0.15	4.89 ± 0.15	4.69 ± 0.14	4.70 ± 0.14	4.65 ± 0.14	4.63 ± 0.14	4.56 ± 0.14			
	Biodegradable	4.93 ± 0.15	4.91 ± 0.15	4.88 ± 0.15	4.62 ± 0.14	4.60 ± 0.14	4.56 ± 0.14	4.52 ± 0.14	4.51 ± 0.14			
Acidity	Plastic	3.68 ± 0.11 ^D,a^	3.87 ± 0.12 ^C,D,a^	3.80 ± 0.11 ^C,D,a^	4.03 ± 0.12 ^C,a^	4.23 ± 0.13 ^B,a^	4.30 ± 0.13 ^A,B,a^	4.33 ± 0.13 ^A,B,a^	4.40 ± 0.13 ^A,a^	<0.001	<0.001	n.s.
	Paper-based	3.67 ± 0.11 ^b^	3.60 ± 0.11 ^b^	3.70 ± 0.11 ^b^	3.70 ± 0.11 ^b^	3.80 ± 0.11 ^b^	3.81 ± 0.11 ^b^	3.99 ± 0.12 ^b^	4.00 ± 0.12 ^b^			
	Compostable	3.63 ± 0.11 ^b^	3.72 ± 0.11 ^b^	3.80 ± 0.11 ^b^	3.79 ± 0.11 ^b^	3.95 ± 0.12 ^b^	3.99 ± 0.12 ^b^	4.03 ± 0.12 ^b^	3.99 ± 0.12 ^b^			
	Biodegradable	3.60 ± 0.11 ^b^	3.53 ± 0.11 ^b^	3.68 ± 0.11 ^b^	3.75 ± 0.11 ^b^	3.93 ± 0.12 ^b^	3.88 ± 0.12 ^b^	3.99 ± 0.12 ^b^	4.13 ± 0.12 ^b^			

Means with different capital letters in the same row or small letters in the same column differ statistically according to Tukey’s test (*p* ≤ 0.05), n.s.: not significant.

**Table 4:** The regression coefficient of the Avrami model.

Packaging	F_∞_	t_1/2_	n	k	R^2^
Plastic	1092.16	0.36	1.50	3.21	0.96
Paper-based	1061.88	0.35	1.44	3.07	0.90
Compostable	1516.37	0.42	1.93	3.64	0.97
Biodegradable	916.78	0.33	1.39	3.19	0.98

F_∞_: final firmness; t_1/2_: crystallization half-time; n: Avrami exponent; k: rate constant; R^2^: correlation coefficient

**Table 5:** Significance levels from two-way ANOVA for the sensory attributes of croissants packed using different films during storage.

Sensory Attributes	Packaging	Storage Time	Packaging × Storage Time
Crust colour	n.s.	n.s.	n.s.
Crumb colour	n.s.	≤0.0001	n.s.
Flaking structure	n.s.	n.s.	n.s.
Overall odour	≤0.0001	≤0.0001	n.s.
Alcohol odour	≤0.0001	≤0.0001	n.s.
Softness	≤0.0001	≤0.0001	≤0.0001
Elasticity	≤0.0001	≤0.0001	≤0.01
Oiliness	n.s.	≤0.0001	n.s.
Stickiness	n.s.	≤0.0001	≤0.05
Sweetness	≤0.001	≤0.0001	n.s.
Overall flavour	≤0.001	≤0.0001	n.s.
Butter flavour	≤0.05	≤0.0001	n.s.
Vanilla flavour	≤0.001	≤0.0001	n.s.
Consistency	≤0.0001	≤0.0001	≤0.001
Softness in the mouth	≤0.0001	≤0.0001	≤0.0001
Adhesiveness	≤0.01	n.s.	≤0.05
Swallowability	≤0.0001	n.s.	≤0.05

n.s.: not significant.

**Table 6:** Predicted shelf-life based on 50% of consumers’ rejection of croissants sealed with different films, and the corresponding values of physiochemical parameters.

Packaging	Shelf-Life (Days)	Moisture (%)	Water Activity	Firmness (N)	pH	Acidity
Plastic	169	17.96	0.809	1224.04	4.48	4.47
Paper-based	142	17.37	0.800	1079.95	4.62	3.91
Compostable	109	17.44	0.789	1284.91	4.69	3.91
Biodegradable	185	17.91	0.801	1063.93	4.38	4.15

## Data Availability

The original contributions presented in the study are included in the article and Supplementary Materials, further inquiries can be directed to the corresponding author.

## Supplementary Materials

The following supporting information can be downloaded at: https://www.mdpi.com/article/10.3390/foods13091390/s1, Figure S1: Effect of storage time on moisture (a), water activity (b), acidity (c), pH (d) and firmness (e) in croissants sealed within different packaging materials.; Table S1: Sensory descriptors and average scores assigned in descriptive sensory analysis for croissants sealed with different films during 150 days of storage.
