# Supplementary material for: A Sensory Shelf-Life Study for the Evaluation of New Eco-Sustainable Packaging of Single-Portion Croissants
Source: Foods. 2024 Apr 30;13(9):1390. doi: 10.3390/foods13091390 (PMC11083942; doi:10.3390/foods13091390)
Supplement: Supplementary file 1 [file foods-13-01390-s001.zip › foods-2969576-supplementary.pdf]

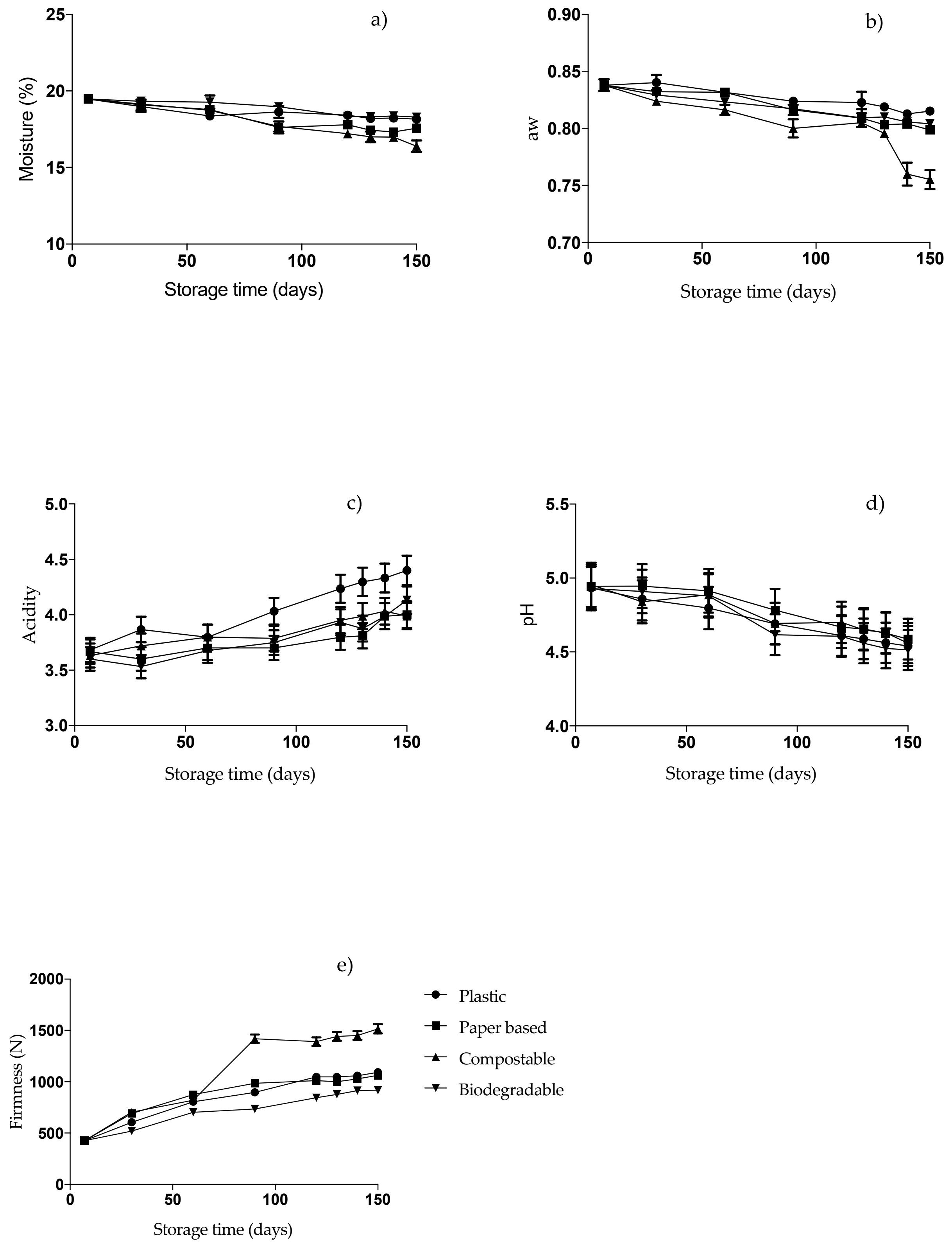

Figure S1. Effect of storage time on moisture (a), water activity (b), acidity (c), pH (d) and firmness (e) in croissants sealed within different packaging materials.

Table S1. Sensory descriptors and average scores assigned in descriptive sensory analysis for croissants sealed with different films during 150 days of storage

Days	Packaging	Crust color	Crumb color	Flaking structure	Overall odor	Alcol odor	Softness	Elasticity	Oiliness	Stickiness	Sweetness	Overall flavor	Butter flavor	Vanilla flavor	Consisten cy	Softness in the mouth	Adhesive ness	Swallowabil ity
7	Plastic	61.7	50.5^{A}	50.1	66.8^{aA}	40.3^{bB}	65.0^{aA}	58.0^{aA}	45.2^{A}	35.9^{A}	63.5^{aA}	64.0^{abA}	48.8^{aA}	44.0^{abAB}	37.9^{bB}	53.9^{bA}	35.0^{ab}	48.8^{bc}
	Paper-based	60.8	47.2	45.3	63.8^{a}	44.4^{a}	56.0^{b}	43.8^{b}	45.6	36.3	66.6^{a}	64.7^{a}	49.2^{ab}	40.6^{b}	35.8^{ab}	53.3^{b}	40.2^{a}	44.1^{ab}
	Compostable	66.4	50.7	46.4	61.2^{b}	45.9^{a}	53.6^{c}	54.7^{b}	41.6	33.6	61.0^{b}	64.3^{b}	46.9^{b}	43.6^{b}	39.8^{a}	50.4^{c}	39.0^{ab}	48.3^{c}
	Biodegradable	66.0	54.1	56.5	61.7^{a}	41.4^{b}	65.0^{a}	55.4^{a}	43.4	40.0	66.2^{a}	63.3^{a}	47.9^{a}	44.8^{a}	33.4^{c}	58.7^{a}	37.9^{b}	50.5^{a}
30	Plastic	59.1	46.6^{AB}	45.7	64.9^{A}	38.8^{B}	55.7^{B}	52.8^{B}	33.2^{B}	27.9^{B}	59.2^{BCD}	$62.3A^{BC}$	42.0^{BCD}	42.5^{BC}	40.1^{B}	51.5^{A}	39.7	52.9
	Paper-based	55.1	47.1	47.4	63.0	41.6	49.2	44.1	36.9	30.2	60.7	62.4	43.2	40.5	36.4	53.5	39.6	57.4
	Compostable	55.8	44.4	41.8	60.2	44.9	45.9	44.7	36.2	30.5	56.4	60.4	41.9	37.6	39.2	53.3	39.9	51.9
	Biodegradable	61.6	48.3	54.9	67.0	35.5	57.3	51.9	36.0	33.8	61.3	63.0	42.4	39.6	32.0	60.1	36.5	54.7
60	Plastic	55.1	44.6^{AB}	51.6	58.3^{B}	39.4^{B}	47.3^{B}	38.7^{D}	27.6^{BC}	22.8^{CD}	57.9^{BCD}	57.1^{D}	44.6^{BC}	37.0^{CD}	41.2^{B}	53.9^{A}	33.5	48.1
	Paper-based	52.5	43.3	45.0	59.5	44.1	47.3	37.5	37.8	23.2	59.9	56.6	42.8	37.5	42.4	48.7	36.2	51.8
	Compostable	56.3	41.9	50.7	56.6	39.0	43.5	35.5	31.2	24.2	56.4	56.6	43.8	38.1	42.1	53.3	34.1	52.3
	Biodegradable	59.3	46.6	51.9	58.3	39.5	55.7	46.9	29.4	22.7	62.2	59.5	44.3	41.3	31.1	57.8	33.8	55.2
90	Plastic	64.9	51.1^{AB}	57.2	58.8^{B}	49.5^{A}	49.9^{B}	45.3^{CD}	33.4^{B}	19.5^{CDE}	62.0^{AB}	58.2^{AB}	43.8^{AB}	43.2^{A}	53.3^{A}	53.6^{A}	36.1	47.6
	Paper-based	60.8	44.5	52.2	56.2	49.6	54.4	43.6	35.1	24.1	63.5	64.6	45.4	45.4	54.3	51.5	40.9	52.2
	Compostable	61.1	46.2	46.5	56.5	53.2	46.3	39.6	36.1	20.4	59.6	63.8	46.4	46.6	57.3	41.6	35.4	43.9
	Biodegradable	62.6	49.5	52.0	62.3	51.4	58.5	42.8	31.5	23.4	66.7	65.8	48.9	51.9	52.8	61.6	35.2	50.4
120	Plastic	58.9	41.9^{B}	52.9	60.1^{B}	51.9^{A}	40.4^{C}	43.7^{CD}	27.3^{D}	27.1^{CD}	58.3^{ABC}	60.1^{BCD}	43.3^{D}	39.4^{D}	52.6^{A}	40.2^{B}	37.1	45.8
	Paper-based	58.2	38.3	45.8	54.2	51.8	37.5	37.4	24.9	24.9	62.9	59.2	35.5	33.6	57.7	39.8	32.6	47.5
	Compostable	61.3	39.4	48.6	54.0	56.4	34.2	38.8	24.9	23.7	57.1	56.1	35.2	32.9	52.0	34.3	35.8	42.6
	Biodegradable	60.6	39.6	48.9	59.2	48.2	43.1	47.4	24.4	20.5	63.8	59.3	39.2	35.7	40.4	46.1	36.1	45.0
130	Plastic	61.7	39.3^{AB}	57.9	59.1^{B}	46.3^{A}	41.5^{C}	45.7^{BC}	23.9^{D}	23.4^{DE}	63.3^{ABCD}	59.6^{CD}	41.7^{CD}	39.3^{BCD}	51.1^{A}	41.4^{B}	35.7	48.1
	Paper-based	60.1	41.1	47.7	59.1	53.1	37.7	40.3	27.1	21.5	61.7	60.4	39.7	37.3	53.0	36.3	39.3	51.9
	Compostable	64.3	37.6	48.3	47.2	50.1	35.1	44.4	25.4	20.3	58.1	52.1	36.2	37.8	58.5	34.7	36.3	45.3
	Biodegradable	64.7	42.3	54.1	58.3	46.7	38.7	47.2	25.6	16.3	57.4	60.7	39.7	43.3	52.1	42.7	34.9	50.7
140	Plastic	60.1	38.1^{B}	47.0	55.4^{B}	45.7^{A}	41.7^{C}	45.1^{BC}	27.5^{CD}	27.3^{C}	58.3^{CD}	53.5^{D}	46.7^{BCD}	35.2^{CD}	53.2^{A}	41.7^{B}	36.1	48.3
	Paper-based	60.8	38.5	52.3	56.2	52.7	36.5	43.7	27.8	26.5	59.3	58.5	46.4	35.5	52.9	35.3	37.7	49.7
	Compostable	64.0	38.1	49.1	54.4	52.8	32.9	44.7	28.1	27.8	54.5	55.6	32.6	33.6	58.4	31.9	38.1	50.3
	Biodegradable	63.7	38.3	53.5	55.9	49.5	37.3	48.7	22.5	23.0	59.4	57.5	45.7	42.2	52.5	41.7	31.6	52.3
150	Plastic	62.8	38.4^{B}	49.2	57.8^{B}	50.2^{A}	38.9^{C}	32.7^{E}	33.8^{B}	19.5^{E}	56.9^{D}	59.4^{D}	41.0^{CD}	36.5^{CD}	51.4^{A}	$39.^{4B}$	36.9	45.5
	Paper-based	62.4	38.6	47.7	54.8	49.1	38.6	33.5	32.0	17.5	54.6	55.9	41.5	37.9	55.7	39.8	34.6	50.3
	Compostable	58.9	37.7	46.0	53.3	55.9	32.3	30.9	34.5	19.8	53.8	49.8	41.6	34.8	58.1	31.9	40.2	41.9
	Biodegradable	63.0	42.1	53.1	53.7	50.5	37.0	36.4	30.1	17.8	57.5	59.9	39.1	37.1	52.1	36.7	32.6	51.8
p value	Packaging (P)	n.s.	n.s.	n.s.	≤ 0.0001	≤ 0.0001	≤ 0.0001	≤ 0.0001	n.s.	n.s.	≤ 0.001	≤ 0.001	≤ 0.05	≤ 0.001	≤ 0.0001	≤ 0.0001	≤ 0.01	≤ 0.0001
	Storage (S)	n.s.	≤ 0.0001	n.s.	≤ 0.0001	≤ 0.0001	≤ 0.0001	≤ 0.0001	≤ 0.0001	≤ 0.0001	≤ 0.0001	≤ 0.0001	≤ 0.0001	≤ 0.0001	≤ 0.0001	≤ 0.0001	n.s.	n.s.
	P x S	n.s.	n.s.	n.s.	n.s.	n.s.	≤ 0.0001	≤ 0.01	n.s.	≤ 0.05	n.s.	n.s.	n.s.	n.s.	≤ 0.001	≤ 0.0001	≤ 0.05	≤ 0.05

Means with different capital letters in the same column or small letters in the same row differ statistically according to Tukey's test ($p \leq 0.05$)
